# Quantity Effect of Radial Cracks on the Cracking Propagation Behavior and the Crack Morphology

**DOI:** 10.1371/journal.pone.0098196

**Published:** 2014-07-21

**Authors:** Jingjing Chen, Jun Xu, Bohan Liu, Xuefeng Yao, Yibing Li

**Affiliations:** 1 State Key Laboratory of Automotive Safety & Energy, Department of Automotive Engineering, Tsinghua University, Beijing, PR China; 2 School of Aerospace, Tsinghua University, Beijing, PR China; University of Zurich, Switzerland

## Abstract

In this letter, the quantity effect of radial cracks on the cracking propagation behavior as well as the circular crack generation on the impacted glass plate within the sandwiched glass sheets are experimentally investigated via high-speed photography system. Results show that the radial crack velocity on the backing glass layer decreases with the crack number under the same impact conditions during large quantities of repeated experiments. Thus, the “energy conversion factor” is suggested to elucidate the physical relation between the cracking number and the crack propagation speed. Besides, the number of radial crack also takes the determinative effect in the crack morphology of the impacted glass plate. This study may shed lights on understanding the cracking and propagation mechanism in laminated glass structures and provide useful tool to explore the impact information on the cracking debris.

## Introduction

Laminated glass panels now are widely equipped in various architectures and mechanical devices serving as engineering bearing and protective structures [1], [2]. Therefore, mechanical properties, especially those under dynamic loadings are of prior interest. PVB (Polyvinyl Butyral) laminated glass is the most widely used polymer-glass sandwiched composite material in buildings and automotive industries [3], [4] and has been receiving heated attentions in investigation of its dynamic mechanical behaviors, such as energy absorption ability [5], [6], damage mechanism [7] and cracking morphologies [8], [9].

Numerous studies addressing the behavior of in-plane crack propagation on the brittle plate under dynamic loading are available [10]–[13]. More emphasizes are put in studying the relationship between the dynamic stress intensity factor and single crack velocity [14], [15]. However, multiple cracks problem which often occurs in real-world engineering applications, has received scant experimental attention at best [16]–[18]. Astrom and Timonen [19] have found three types of cracks on brittle plates by a localized transverse impact resulting from different impact conditions. Recently, some cracking morphology investigations are also of great interest to researchers which models the relation between crack number on mono-layer plate material and impact parameters [20].

In this study, we focus on the multi-crack behavior on the laminated glass material. The effect of the radial crack number on their own crack propagation velocity as well as the crack generation on the other glass layer is thoroughly investigated. Firstly, the radial crack velocity on the backing glass layer with different crack numbers is calculated. A theoretical model from the perspective of energy conversion is established to depict the cracking process of backing glass layer and elucidate the fundamental reason that causes the variation of the crack velocity. Further, the cracking morphology of the impacted glass layer affected by the radial crack number of backing glass layer is investigated through large quantities of experiments. The total crack length for both two crack patterns on the impacted glass layer is calculated to study the capability of energy absorption of laminated glass influenced by the radial crack number on the backing glass layer.

## Experiment

**Figure 1:**
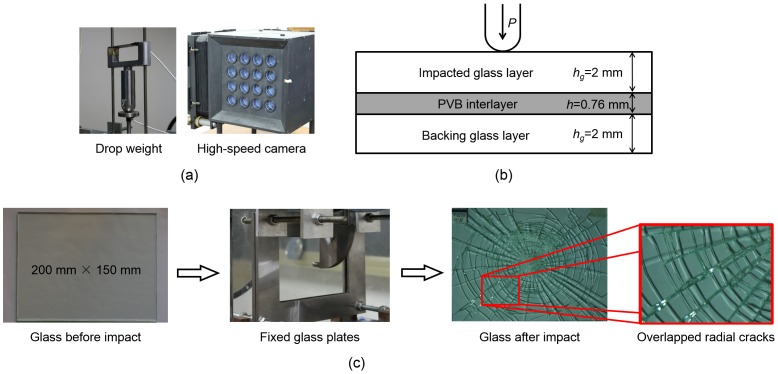
(a) Schematic illustration of the experiment setup. (b) Schematic of the impact process to the layered glass. (c) View of glass sample before and after impact.

The high-speed photography system in combination with the drop-weight platform used in the reference [21], [22] is employed in the dynamic fracture experiment in this letter. The drop-weight tower (Figure 1(a)) sliding along the two standing poles is used to provide the impact energy, whose maximum height is 1000 mm while the mass of the drop-weight in this experimental investigation is 2 kg. A hemispherical impactor is fixed to impact the plates perpendicularly at their center (Figure 1(c)). The high-speed photography system is used to capture the crack propagation process on the glass plates. The laminated glass used in our experiments is exactly the windshield glass material widely employed in the automotive industry [6], which is cut into 200 mm×150 mm large. The plate is consisted of a PVB (Polyvinyl Butyral) interlayer sandwiched by two brittle glass sheets, fixed between two frames in which the rubber pad is added by each side to receive the clamping force more evenly (Figure 1(c)).

Prior to impact, the drop-weight is suspended and fixed through electromagnet at certain height. Once the drop-weight tower slides down and contacts the glass surface, a trigger signal is generated by means of a time sequence controlled circuit. The signal firstly triggered the delay controller, causing the orderly ignition of the 16 spot lights under the ultra-high voltage. Meanwhile, the 16 images containing the crack information were obtained. All the experiments were conducted in the temperature range of 20–25°C and in the humidity range of 20–30% [21].

## Results

**Figure 2:**
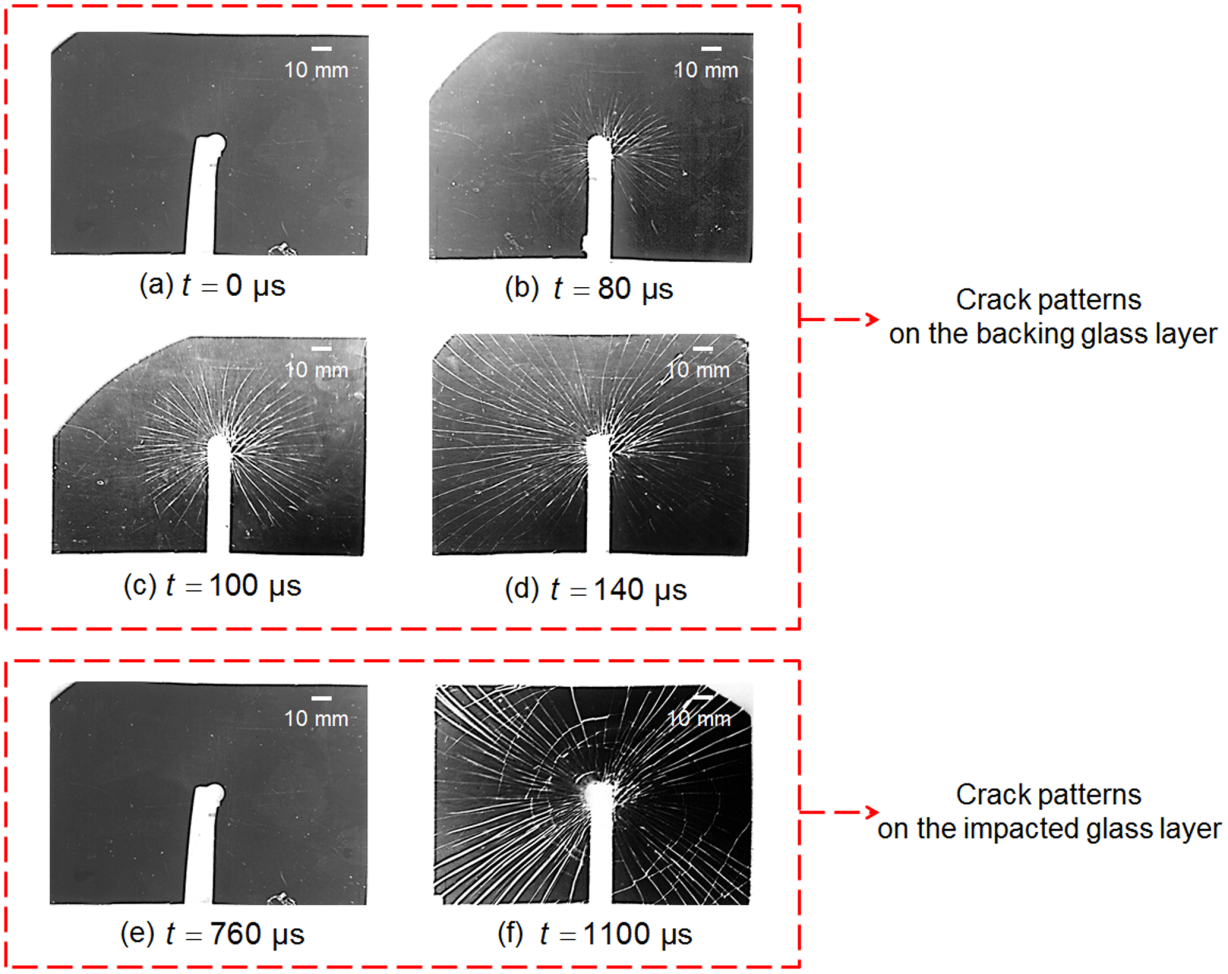
Images depicting the crack growth on the two glass layers (*V* = 3.7 m/s).

The specimen in response to impact speed *V* = 3.7 m/s is chosen for one hundred repeated experiments by considering the intrinsic stochastic flaws in glass laminated samples. A series of images depicting the crack growth on both glass plates are recorded at the set time intervals. Typical crack propagation process is shown in Figure 2. The glass plate directly contacting with the impactor is defined as “impacted glass layer” while the glass plate on the other side is referred as “backing glass layer”. 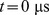 represents the time when impact is triggered. Figure 2(a)–(d) demonstrate the whole cracking process on the backing glass layer where cracks always initiate first, and only radial crack pattern is observed while both radial and circular cracks appear on the impacted glass layer (under the effect of Rayleigh waves, whose propagation is limited to the loaded surface of the solids) long after the cracking of the backing one (Figure 2(e)–(f)) [22]. However, the radial crack number on the backing glass plate varies from 18 to 112 within one hundred repeated experiments, which is quite a large deviation. Further, it is also qualitatively observed that the number of radial cracks have great effect on the later crack generation on the impacted glass layer as well as the propagation characteristics of radial crack on the backing glass layer itself. In the following section, the effect of the different radial crack numbers of backing glass layer would be thoroughly discussed.

## Discussion

### Crack velocity on the backing glass layer

**Figure 3:**
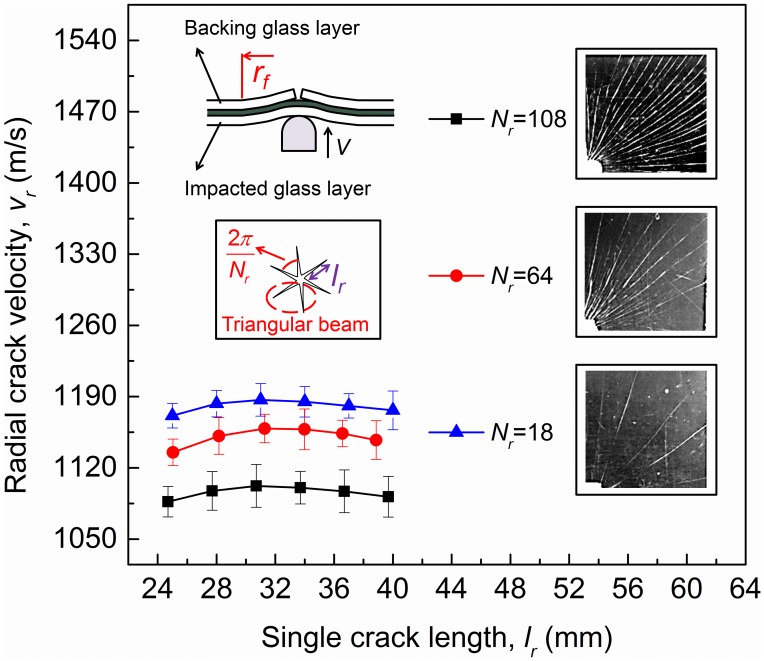
Radial crack velocity vs. crack length with different radial crack number (*V* = 3.7 m/s).

The crack velocity history on the backing glass layer with different radial crack number is calculated separately. All the radial cracks present approximately the same length during the propagation, conforming a circle overall (Figure 2). Therefore, the crack velocity *v_r_* is calculated as the radius increase 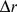 over the time increase 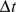, i.e. 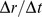. Figure 3 shows the crack velocity variation with the crack length *l_r_* under three selected radial crack numbers (*N_r_* = 18, 64 and 108). Crack propagations far from the boundaries and the center of the glass plate are focused to eliminate the possible boundary effect (i.e. the crack length between 20 mm to 40 mm). Results indicate that the number of radial cracks on the backing glass layer regularly negatively influences cracking propagation velocity themselves, i.e. the crack velocity *v_r_* always decreases with the radial crack number increasing.

During the propagation of the cracks on the backing glass layer, the impacted glass layer remains intact as stated before (Figure 3). However, due to the adhesion by PVB interlayer, the two glass layers have the same radius of deformed region *r_f_* (Figure 3). The bending energy of the impacted glass plate caused by the impact can be estimated by assumption that the backing glass layer is still intact, in which case the two glass plates can be regarded as “single one with 2*h_g_* thickness”. Therefore, the bending energy of impacted glass layer is equal to half the energy in the single glass plate with 2*h_g_* thickness. After the contact between the impactor and the glass plate, kinetic and bending energies balance 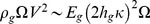 [20], where 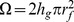 is the volume of deformed region and *V* is the impact speed whose variation can be ignored during such a short period. Thus the curvature 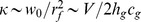, yielding 
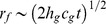
, where 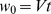 is the indentation and 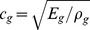 is the sound speed in the glass material. The bending energy of impacted glass plate 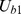 can be expressed as
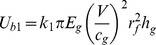
(1)where 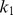 is a coefficient.

Considering the low Young's modulus of PVB material (i.e. 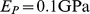), the bending energy of PVB 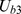 is about 0.001 of that in the glass plate, which has been proved by the finite element simulation with a three-dimensional laminated plate FE model using 1 mm×1 mm surface size element. The boundary condition of the glass plate model is set to be clamped, which is consistent with the real experimental condition. Thus, 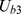 is small enough to be ignored compared to that of 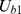.

While for the backing glass layer in the cracking process with crack length *l_r_*, the number of radial cracks *N_r_* on the backing layer has been set and would not change as the cracks extend. Thus the bending energy of the backing glass plate 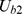 can be estimated by the energy of *N_r_* triangular beams of length *l_r_* with the neglect of transverse bending [20]:
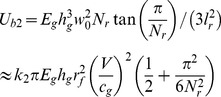
(2)where 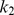 is another coefficient. Therefore, the bending energy of the whole laminated plates 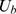 can be estimated as
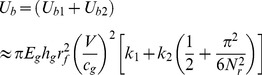
(3)


From the perspective of energy transferring, during the cracking process, the elastic energy of the laminated plates (i.e. the bending energy) caused by the impact is converted into the glass surface energy through the fracture behavior. Here we define the increasing rate of the plates elastic energy as “
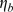
”, and the energy release rate is “
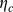
”. Therefore, we have
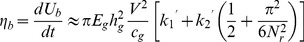
(4)

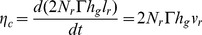
(5)where 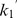 and 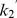 are both coefficients. 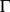 is the glass material fracture (surface) energy. There should be a balance between the “input energy” (i.e. the increasing elastic energy) and the “output energy” (i.e. the fracture energy). Suppose that the proportion between the two parameters 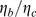, which is named as “energy conversion factor”, indicating the stability of the system or the ability for further crack growth, then the larger proportion would obviously lead to a greater degree of instability for the whole system. Thus for the system of PVB laminated plates in our study, the “energy conversion factor” 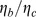 can be expressed as
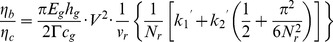
(6)


One may clearly see that the expression of the “energy conversion factor” contains three parts: (1) 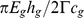 is determined by the material properties (here refers to the glass material); (2) 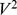 refers to the impact condition; (3) rest of the expression 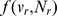 refers to the impact responses of the laminates. Thus the stability of the system is determined together by the material properties, impact condition and the impact response of the material according to Eqn. 6. Previous studies [21], [22] investigating the effect of the impact speed on the crack velocity have demonstrated that, with higher impact speed *V* that increasing the system instability 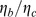, both of the crack velocity *v_r_* and the radial crack number *N_r_* tend to increase as well thus to lower the value of 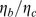 such to maintain the balanced energy input and output in a large extent. Therefore, as the crack number *N_r_* on the backing glass layer decreases under the same impact condition, the value of 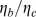 would correspondingly increase due to the result of Eqn. 6, which means the instability of the system is rising. Thus the system tends to response in a way to reduce this instability (i.e. the value of 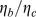) through propagating the cracks at higher speed, improving the value of *v_r_* in Eqn. 6, which is consistent with our experiment results (Figure 3).

### Crack morphology on the impacted glass layer

**Figure 4:**
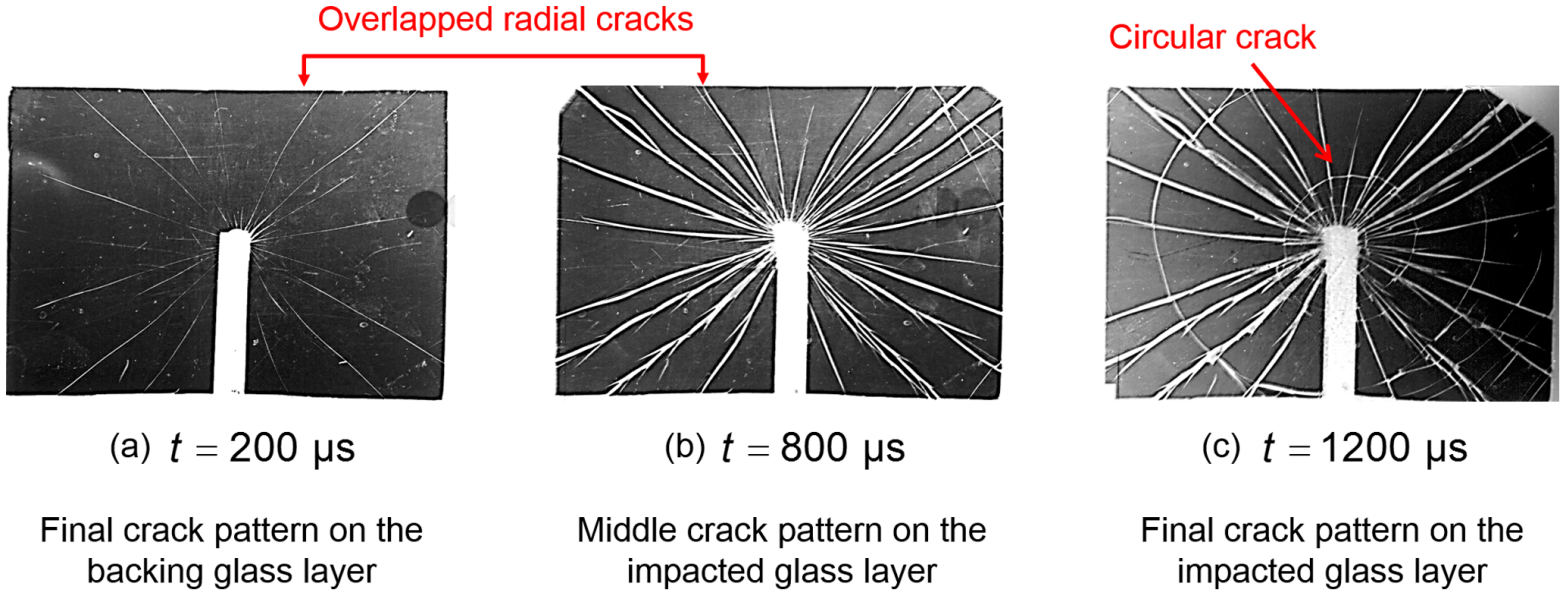
Selected sequence of images depicting the crack patterns on both glass layers at the loading speed *v_d_*  = 3.7 m/s, with PVB thickness *h* = 0.76 mm.

Figure 4 shows the crack morphology on the impacted glass layer where both of the two crack patterns (i.e. radial crack and circular crack) appear long after the cracking of the backing glass plate. For the radial cracks on the impacted layer that are indirectly caused by the stress concentration brought by each generated crack on the backing glass layer as initial flaw [23] (which is also proved by our experiment investigation), they are completely overlapped with the radial cracks on the backing layer (Figure 4). Thus it's obvious that the morphology as well as the number of radial cracks in the impacted glass layer depends completely on that of the backing layer. The radial cracks on the impacted glass initiates from those on the backing glass plate through the depth direction [23].

**Figure 5:**
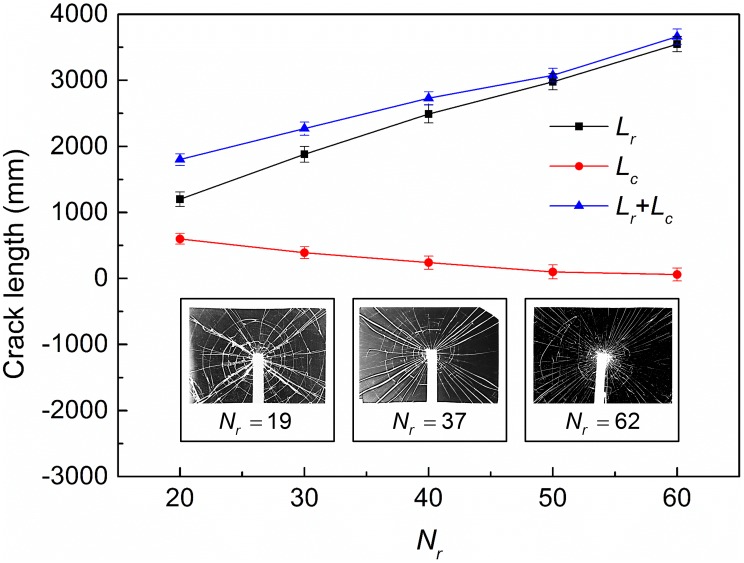
Crack length on the inner glass layer vs. radial crack number (*V* = 2.42 m/s).

Further, specimen in response to lower impact speed *V* = 2.42 m/s is chosen for one hundred repeated experiments. As expected, the radial crack number on the backing glass plate varies from 15 to 65 in these experiments. The total crack length for both two patterns on the impacted glass layer is calculated with different crack numbers on the backing glass layer (Figure 5). *L_r_* and *L_c_* are defined as the total radial and circular crack length on the impacted glass layer respectively. Thus (*L_r_*+*L_c_*) refers to the total crack length on the impacted glass plate. The radial crack length linearly increases with crack number on the backing glass layer due to the uniform growth of all cracks while the variation trend of circular crack length is decreasing (Figure 5). Circular cracks always initiate long after the completed propagation of radial cracks on the impacted glass [24] (Figure 4). Thus, before the initiation of circular cracks, the laminated plates can be regarded as *N_r_* triangular beams with thickness 2*h_g_* and length *l_0_* (which can be estimated as the side length of specimen) since the radial cracks on the two glass plates are completely overlapped (Figure 4). The bending energy 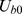 of the plates is
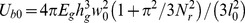
(7)


In the case with less radial crack number *N_r_*, the bending energy would increase, resulting in stronger stress concentration in the radial direction, which makes the later generation of circular cracks easier. In addition, taking the law of energy conservation into account, the total fracture energy on the whole laminated plates can be estimated as 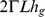, where *L* is the total crack length on the two glass plates. Thus the fracture energy released on two glass plates can be estimated by calculating the crack length on each plate. For the plates with shorter radial crack length, leaving more residual energy, it will benefit the generation of circular cracks as well.

Although the increase of circular crack number contributes positively to the energy absorption of the laminated plates, the effect is much less compared with the radial cracks (Figure 5). As the radial crack morphology on the impacted glass is directly determined by that on the backing glass layer, it is safe to conclude that the backing glass plate is the key that decides the fracture morphology and further the capability of energy absorbing of the whole laminated structure such that the material selection and structure design for the backing layer is extremely important for impact protection.

## Concluding Remarks

In this letter, the number effect of radial cracks on the propagation behavior of their own as well as its decisive influence on the cracking morphology of the other glass plate in the laminated structures has been experimentally investigated. The cracking process and the final crack morphology on the two glass layers have been captured using the high-speed photography system in combination with the drop-weight platform. The propagation velocity of radial cracks on the backing glass layer is proved to be regularly influenced by their amount under the same impact conditions. A theoretical model is established depicting the energy conversion during the cracking process of backing glass layer. The “energy conversion factor” is suggested, illustrating the fundamental reason that causes the variation of the crack velocity, which is fitted well with the experiment results. Quantities of experiments also prove that the radial crack number on the backing layer takes determinative effect in the crack morphology of the other glass plate. The length of two crack patterns on the impacted glass layer varies differently with the radial crack number *N_r_*. However, the fracture energy of the whole laminated plates is mainly determined by the radial crack length, which means that the radial crack number generated first on the backing glass plate is the key that decides the capability of energy absorbing of the whole laminated structure. The PVB interlayer effect on the cracking behavior will be discussed elsewhere. This study has focused on the in-plane interaction of multiple cracks during their propagation process as well as the inter-plate interaction of laminated glass on the crack morphology, which may provide a solid foundation for further investigations on the dynamic facture behavior of the material with layered structures.
